# Valorization of camelina oil to biobased materials and biofuels for new industrial uses: a review

**DOI:** 10.1039/d2ra03253h

**Published:** 2022-10-06

**Authors:** Muhammad Arshad, Amar K. Mohanty, Rene Van Acker, Rachel Riddle, Jim Todd, Hamdy Khalil, Manjusri Misra

**Affiliations:** Department of Plant Agriculture, Bioproducts Discovery & Development Centre, Crop Science Building, University of Guelph Guelph Ontario N1G 2W1 Canada mohanty@uoguelph.ca; School of Engineering, Thornbrough Building, University of Guelph Guelph Ontario N1G 2W1 Canada mmisra@uoguelph.ca; Department of Plant Agriculture, University of Guelph Guelph ON N1G 2W1 Canada; Department of Plant Agriculture, University of Guelph Simcoe Research Station, 1283 Blueline Road Simcoe Ontario N3Y 4N5 Canada; Ontario Ministry of Agriculture, Food and Rural Affairs Simcoe Research Station, 1283 Blueline Road, Simcoe ON N3Y 4N5 Canada; The Woodbridge Group 8214 Kipling Avenue Woodbridge ON L4L 2A4 Canada

## Abstract

Global environmental pollution is a growing concern, especially the release of carbon dioxide from the use of petroleum derived materials which negatively impacts our environment's natural greenhouse gas level. Extensive efforts have been made to explore the conversion of renewable raw materials (vegetable oils) into bio-based products with similar or enhanced properties to those derived from petroleum. However, these edible plant oils, commonly used for human food consumption, are often not suitable raw materials for industrial applications. Hence, there is an increasing interest in exploring the use of non-edible plant oils for industrial applications. One such emerging oil seed crop is *Camelina sativa*, generally known as camelina, which has limited use as a food oil and so is currently being explored as a feedstock for various industrial applications in both Europe and North America. Camelina oil is highly unsaturated, making it an ideal potential AGH feedstock for the manufacture of lower carbon footprint, biobased products that reduce our dependency on petroleum resources and thus help to combat climate change. This review presents a brief description of camelina highlighting its composition and its production in comparison with traditional plant oils. The main focus is to summarize recent data on valorization of camelina oil by various chemical means, with specific emphasis on their industrial applications in biofuels, adhesives and coatings, biopolymers and bio-composites, alkyd resins, cosmetics, and agriculture. The review concludes with a discussion on current challenges and future opportunities of camelina oil valorization into various industrial products.

## Abstract 2

This review gathers current research work, and strategies for valorization of an emerging non-food camelina oil seed crop into renewable polymers building blocks for industrial applications, current challenges, and future opportunities.

## Graphical abstract

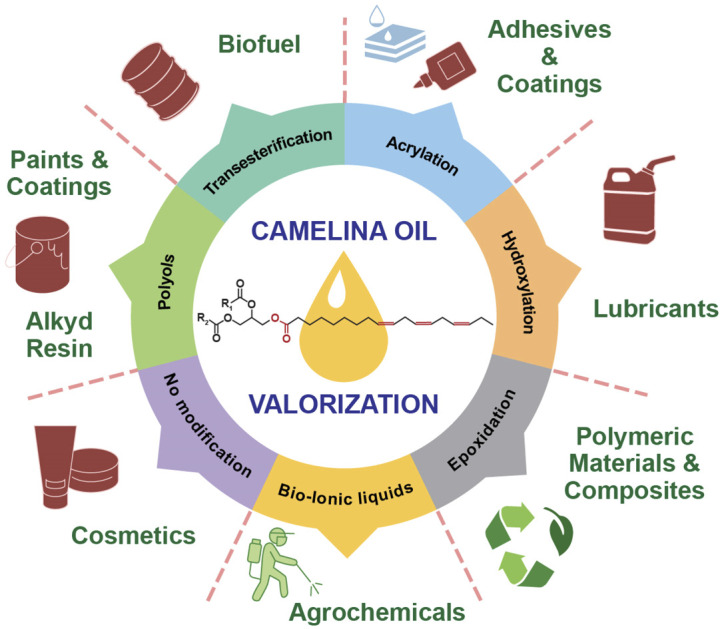

## Introduction

The introduction part of this review provides a brief description of camelina oil, and a comparison of its production, yield, and composition to other traditional plant oils. The Brassicaceae *Camelina sativa*, generally known as camelina, is a winter or spring annual oil seed plant. It is currently garnering renewed attention by both research and industry in Europe and North America^1,2^ due to agronomic qualities that differ from other traditional oil seed crops. These include tolerance to cold weather, a reduced requirement for agricultural inputs, a short growing season (85 to 100 days), resistance to common pathogens and pests, the ability to grow in soils with low fertility levels and good tolerance to semi-arid environmental conditions.^3–5^ These characteristics are very uncommon for an oil seed crop,^6,7^ with rapeseed, canola, soybean, and sunflower all having higher requirements for fertilizer, pesticide protection and water.^8^ Camelina is grown mainly as a nonfood crop in different regions of Europe and Northern America. The use of cold-pressed oil as a food ingredient is allowed in Canada^9^ where camelina meal is also registered as a feed for broiler and layer chickens.^10^

**Fig. 1:**
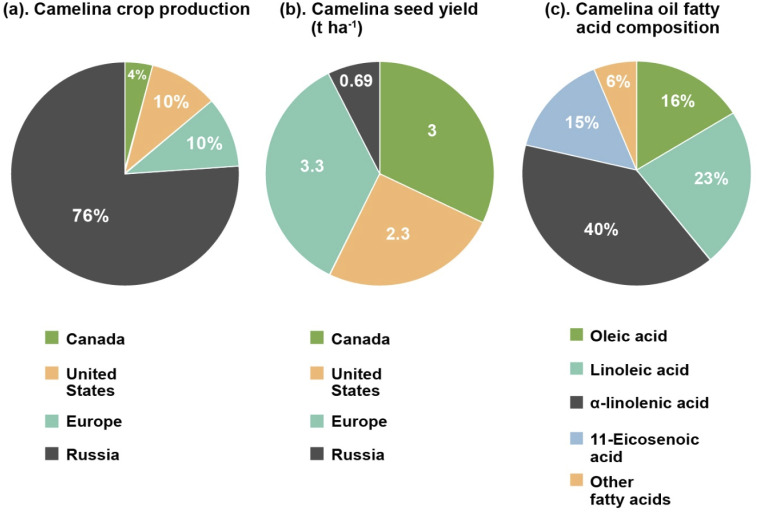
Comparison of camelina annual crop production^11–13^ (a), and camelina seed yield^15–18^ (b) by most of the camelina growing countries (Canada, United States, Europe, and Russia), and its approximate fatty acid composition highlighting major fatty acids contents^24,25^ (c).

As a minor crop, global camelina cultivation is low. Nowadays, it is primarily produced in North America, Russia and Europe for commercial purposes. In Canada approximately 4050 ha (hectares) were cultivated in 2020 (reported by Smart Earth Camelina Corporation), with the majority of those being planted in Saskatchewan.^11^ In 2007, production in the United States was approximately 9700 ha, centered mostly in Montana.^12^ This is similar to the 10 000 ha. Grown in Europe. Russia is the largest camelina producer having an estimated 75 600 ha. In cultivation in 2019 (ref. 13) (Fig. 1a). Variations in global camelina yields have been attributed to weather conditions, cultivar grown and other parameters.^14,15^Fig. 1b illustrates the yield of camelina seed from different parts of the world. In Canada, yield of camelina seed has been observed to be approximately 3 t ha^−1^,^16^ while in United State it is 2.3 t ha^−1^.^17^ In Russia and Europe, camelina yields are reported to be around 0.69 t ha^−1^ and 3.3 t ha^−1^ respectively.^15,18^
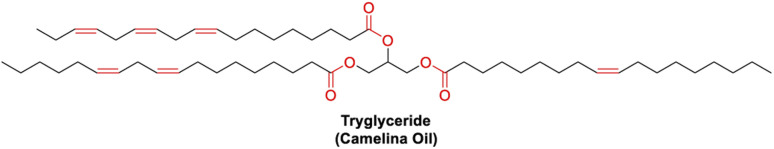


A comparison of the oil and protein content of camelina seeds to other popular edible and non-edible oil seed crops such as soybean (*Glycine max* L.), flax (*Linum usitatissimum* L.), sunflower (*Helianthus annuus* L.), canola oil (*Brassica napus* L.), castor oil (*Ricinus communis* L.) and jatropha oil (*Jatropha curcas* L.) is depicted in Table 1. It is obvious from the values given in Table 1, that camelina seed contains similar or higher levels of oil (30–49%) than the other oil seeds and is second only to canola^19–21^ in protein content (24–31%). Table 1 also shows that, although camelina oil yields vary (106–907 l ha^−1^), they can be higher than the oil yield of sunflower (500–750 l ha^−1^), soybean (247–562 l ha^−1^), and jatropha (741 l ha^−1^), but lower than the oil yield of flax (870–1653 l ha^−1^), castor (1250–2500 l ha^−1^), and canola (1426–1796 l ha^−1^). In addition to commonly found components, ω-3 fatty acids, ω-6 fatty acids, phytosterols, tocopherols, and phenolic compounds are also present in camelina seeds.^14,20,22^ Additionally, carbohydrates ranging from monosaccharides to polysaccharides, and bioactive compounds such as phytic acid, glucosinolates, and condensed tannins are also reported in camelina seeds.^23^

**Table 1:** Comparison of camelina seeds, oil, and protein contents, fatty acids, total phenolics and tocopherol contents with other common oil seeds

Oil seeds	Camelina (*Camelina sativa* L.)	Soybean (*Glycine max* L.)	Flax (*Linum usitatissimum* L.)	Sunflower (*Helianthus annuus* L.)	Canola (*Brassica napus* L.)	Castor (*Ricinus communis* L.)	Jatropha (*Jatropha curcas* L.)
Oil contents (%)	30–49	19–22	37–49	20–43	44–53	30–50	37–45
Protein contents (%)	24–31	39–49	19–24	15–21	17–27	32–48	50–62
Oil yield (l ha^−1^)	106–907	247–562	870–1653	500–750	1426–1796	1250–2500	741
Saturated fatty acids (%)	9.1–13.2	16.4–19.3	8.0–9.9	10.7	5.5–8.3	5.40	19.0
Monounsaturated fatty acids (%)	26.0–41.4	17.5–23.1	13.3–20.1	27.5	60.1–78.0	83.35	42.0
Polyunsaturated fatty acids (%)	50.8–66.6	60.1–64.3	71.7–78.5	61.8	15.1–31.0	11.25	39.0
Total phenolics (mg kg^−1^)	990–1536	183–554	88–156	1800–7200	756–1324	<1.0	263–312
Total tocopherols (mg kg^−1^)	410.0–800.0	114.0–361.1	92.6–311.6	153.5–513.3	166–526	605–785	69–182
Ref.	18–20, 22, 24 and 26–28	28–34	27 and 35–40	27, 28 and 41–43	23, 41 and 44–47	48–53	48–52

A comparison of the fatty acid profile (saturated, monounsaturated, and polyunsaturated), total phenolic and tocopherol content of camelina seeds with other common edible and non-edible oil seeds like soybean, flax, sunflower, canola, castor and jatropha is depicted in Table 1.^23^ Camelina seeds are a good source of unsaturated fatty acids (monounsaturated and polyunsaturated), tocopherols, and phenolic compounds as shown by the values given in Table 1. The qualitative analysis of the fatty acid profile of camelina seeds shows the presence of oleic acid, stearic acid, myristic acid, palmitic acid, nervonic acid, palmitoleic acid, linoleic acid, lignoceric acid, α-linolenic acid, γ-linolenic acid, heneicosanoic acid, 10-heptadecenoic acid, 11,14-eicosadienoic acid, 11- eicosenoic acid, erucic acid, and arachidic acids. Of these, oleic acid (14–16%), linoleic acid (15–23%), α-linolenic acid (31–40%), and 11-eicosenoic acid (12–15%) are the main fatty acid components of camelina oil (Fig. 1c).^24,25^

The composition of camelina oil varies in response to factors such as location, environmental conditions, cultivar, agronomic production cycle and the process of oil extraction.^14,15^ Due to the highly unsaturated fatty acid (triglycerides) content (90%) of camelina oil (Table 2), it is reported to have an average of 5.8 double bonds per triglyceride as compared to soybean oil which contains approximately 84% unsaturated fatty acids with a 4.6 degree of unsaturation.^54^

The next section of this review covers the processes used for valorization or derivatization of camelina oil into useful monomers or polymer precursors. Various industrial applications of camelina oil such as biofuels, polymeric materials and composites, adhesives and coatings, cosmetics, and its non-food use are presented. Moreover, current challenges and future perspectives regarding the industrial use of camelina oil are also discussed.

**Table 2:** Chemistry of camelina oil *vs.* other triglycerides

**Triglyceride's definition and structure**
Fatty acids exist mainly as triglycerides, which are tri-esters of a glycerol bonded to three fatty acid molecules particularly having C16 and C18 carbon chain length ranging from C4 to C24. These triglycerides contain reactive sites or functional groups (esters or acids and C=C double bond) which are the main driving force for their valorization^137^
The chemistry of plant oils mainly differs in their fatty acid profiles which are considered a key element for their valorization into biofuels, value-added products, and organic chemicals through various routes
**Composition**
Triglycerides of camelina oil are composed of around 10% saturated fatty acids and up to 90% unsaturated fatty acids with 26.0–41.4% monounsaturated fatty acids and 50.8–66.6% polyunsaturated fatty acids. Of these, oleic acid (14–16%), linoleic acid (15–23%), α-linolenic acid (31–40%), and 11-eicosenoic acid (12–15%) are the main fatty acid components of camelina oil^23^
The presence of high levels of α-linolenic acid, and comparatively low 11-eicosenoic acid amounts make camelina oil unique compared to other traditional oils (soybean, flax, sunflower, and canola oil)

## Valorization of camelina oil for industrial applications

Plant oils are considered renewable and biodegradable and exhibit numerous advantages over petroleum feedstocks. These include their ease of availability and the wide range of possible chemical modifications that can be done to yield useful polymeric starting materials. Particularly, epoxidized plant oils are widely used by industry in applications that include lubricants,^55^ plasticizers,^56^ composites,^57–59^ resins,^60,61^ polyols,^62^ coatings,^63^ adhesives,^64^ and elastomers.^65^ Among traditional oil seed crops, camelina is seen as a promising energy crop due to its high oil content. Overall, valorization of the entire camelina biomass would provide worthy products such as oil and meal, food and feed, biofuels, and other high value-added industrial products. The high unsaturated fatty acid content and unique characteristics of camelina oil compared to other common seed oils makes it an attractive potential feedstock for various industrial applications, as these unsaturated fatty acids can be easily functionalized into useful polymer building blocks or prepolymers (Fig. 3).

**Fig. 2:**
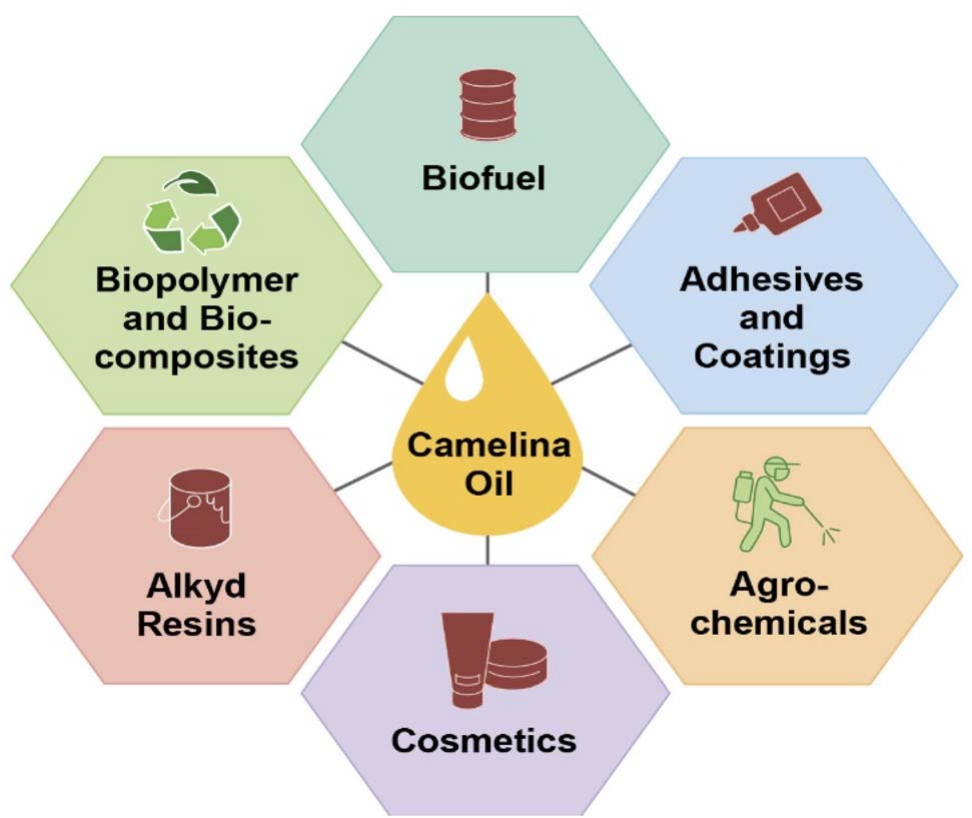
Valorization of camelina oil triglycerides into industrial products such as biofuel,^1,77,85,87^ adhesives and coatings,^54,114,115^ agrochemicals,^129^ cosmetics,^130^ alkyd resin,^120,121^ biopolymers, and bio-composites^106–109^ through various chemical means.

*Camelina sativa* is being used in a wide range of industrial, nutraceutical, and biomedical products and as an animal feed. More specifically, *Camelina sativa* is widely used as a biofuel including renewable jet fuel, green diesel, and biodiesel. It is also being used in processed foods for human consumption.^66–68^ Use of camelina oil for edible purposes is very limited, hence it can serve as a potential non-food competing oil crop to fulfill the demand for biofuel.^69^ Due to the distinct characteristics of camelina oil, it has also demonstrated applications in the area of agriculture, nutrition, and medicine.^70^ Major industrial applications of camelina oil are highlighted in Fig. 2 and elaborated on in the next sections.

### Biofuel production

The continued use of conventional, non-renewable fuel has a large negative environmental impact because of the resulting high emissions of greenhouse gases. Due to this, an increased interest has been observed for the production of renewable fuels such as biodiesel, ethanol, and bio-jet fuel as oils from renewable resources which would reduce the carbon footprint significantly compared to petroleum fuels and conventional diesel.^71–76^ In this regard, several renewable feedstocks such as seed oils, animal fats, algae, and numerous low value waste materials (greases, cooking oil, and soaps) are being investigated as feedstocks for biofuel production.^28^ Not all oilseeds are suitable feedstocks for biofuel production. High seed yield and oil content, unique fatty acid profile, uniform seed maturation rate and adaptability to local growing environment are some of the desirable characteristics of vegetable oils for biofuel preparation.^28^

Moreover, usage of non-edible oilseed crops for biofuel production is highly desirable to maintain a balance with the production of food. In this regard, camelina oil is one the most suitable nonfood oils (in terms of human consumption) when compared to other common oil seeds and is considered an attractive renewable feedstock for biofuel production. In recent times, an increased interest has been reported in the literature for the utilization of camelina oil as a new renewable feedstock for biodiesel and jet fuel production.^77–80^

**Fig. 3:**
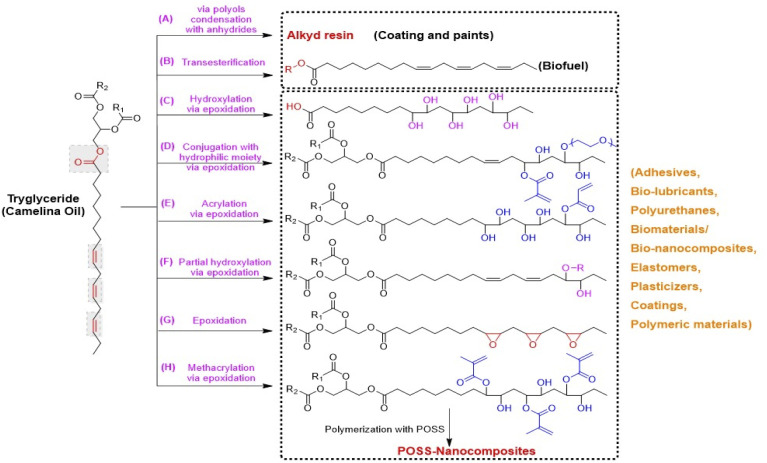
Chemical derivatives/modifications of camelina oil triglycerides into polymer precursors or monomers *via* different chemical routes, (A) alkyd resin by the condensation of polyols, anhydrides or dicarboxylic acids with fatty acids,^113^ (B) biofuels by transesterification of triglycerides into free fatty acid methyl or ethyl esters,^79^ (C) dihydroxy fatty acids *via* epoxidation and ring opening of epoxy groups of triglycerides with water in the presence of acid catalyst,^103^ (D) hydrophilic monomers from triglyceride epoxidation followed by its partial grafting with methacrylic groups and hydrophilic polyethylene glycol units respectively,^99^ (E) an acrylic polyol-a fatty acid monomer with one acrylate functional group *via* triglyceride epoxidation, followed by its partial grafting with acrylic acid and then dihydroxylation with water,^114^ (F) a polyol monomer form controlled partial epoxidation of triglyceride followed by its hydroxylation with an alcohol,^113^ (G) epoxidation of triglyceride with hydrogen peroxide and formic acid in the presence of acid catalyst,^113^ (H) methacrylated fatty acid monomer obtained by epoxidation and subsequent methacrylation of triglyceride.^106^

The free fatty acid methyl or ethyl esters used to manufacture biofuels are typically produced by the transesterification of trigylcerides (Fig. 3). Various reports are published on the transesterification of camelina oil with alcohols (methanol or ethanol) in the presence of a catalyst to produce long chain fatty acid methyl or ethyl esters. These esters can be used to generate biofuels which serve as an effective and environment friendly (low emissions) alternative energy source in place of conventional transport fuels.^81,82^

When compared to mineral diesel fuel, camelina derived biodiesel has improved performance in terms of power, a 50% reduction in visual smoke, lower CO emissions and a decrease in the discharge of toxic components such as NO_*x*_ from the combustion gases.^83,84^ Moreover, biodiesel derived from camelina oil has demonstrated better environmental performance when compared to rapeseed and soybean oil when the impact of changes in land use are considered.^85,86^ Although biodiesel made from camelina oil has many positive characteristics, it still has number of drawbacks including a high iodine value, high Cloud Filter Pour Point (CFPP), high Conradson Carbon Residue (CCR) values, and a lower oxidation stability.^85,87,88^ Thus, the use of camelina oil methyl esters alone is not suitable as a fuel. Mixtures of camelina oil methyl esters (50–60%) with those derived from animal fats (pork lard or used frying oil) have been shown to produce a fuel that meets the desired iodine value standards. Moreover, addition of industrial antioxidants, *e.g.*, ionol, can be used to adjust the oxidation stability.^87,88^

Numerous processes including hydro-processing or hydro-deoxygenation, transesterification, and cracking in the presence of a catalyst^76^ are used for the conversion of vegetable oils into biofuel, as direct use of these oils as biodiesel is not suitable due to incompatibility with engines. Hydro-deoxygenation is a catalytic reaction process with hydrogen for the removal of oxygen under high pressure and moderate temperature. In this process, high pressure hydrogen reacts with bio-oil to produce water and linear alpha olefins (LAOs) in the presence of a catalyst.^89^ Linear alpha olefins produced from vegetable oils exhibit higher added value as compared to fuels. Unsaturated fatty acids can be decarboxylated in the presence of a homogenous catalyst to yield unsaturated hydrocarbons.^90,91^ Moreover, ethenolysis of vegetable oils also provide LAOs,^92,93^ as long chain linear alpha olefins are considered an interesting constituent of lubricants and surfactants.

On a commercial scale, transesterification is the typical process used to produce biodiesel from vegetable oils, where the chemical conversion of triglycerides into alkyl esters occurs by reaction with alcohols in the presence of a catalyst. The resulting fatty acid methyl or ethyl esters alone are not fully compatible with conventional diesel engines due to their inherent high oxygen content, hence blending of these biodiesels with petroleum fuels is usually done.^94^ Moreover, these plant derived fatty acid alkyl esters have shown poor cold flow properties such as high pour point, cloud point and cold filter plugging point.^91^

Catalytic or thermal decomposition of triglyceride structures into smaller alkanes, alkenes, and fatty acids is carried out by a process called cracking.^95^ Conventional cracking catalyst such as mesoporous aluminosilicates and zeolites are used by the petroleum industry, and could be considered for use in the processing of vegetable oils.^96^ However, these processes are highly unselective and result in the formation of a large range of oxygenates and hydrocarbons.^95^

Nestor *et al.* (2012), reported on the production of camelina oil derived biodiesel *via* methanolic transesterification using a base catalyst and compared it with biodiesel obtained from other oil feedstocks such as mustard, coconut, canola, soybean, palm, and sunflower. Fuel properties, including the fatty acid profiles of all vegetable oils were investigated according to the ASTM D6751 standard. The camelina oil derived biodiesel fatty acid profile was found to be 48–50% polyunsaturated, 37–40% monounsaturated and 10–12% saturated. Certain properties, for instance flash point, oil stability index, cloud point, kinetic viscosity at 40 °C and cold filter plugging point of camelina biodiesel were observed to be comparable to sunflower derived biodiesel. However, the higher content of n-3 fatty acids in camelina oil after biodiesel production accounted for its high potential to produce a coke during combustion, high distillation temperature and poor oxidative stability.^97^

Another study by Zaleckas *et al.* (2012) investigated the physical and chemical properties of biodiesel derived from camelina oil and reported a high iodine value and poor oxidation stability of its methyl fatty acid esters. This led to the recommendation that when used as a biodiesel, it be blended with 40 to 50% methyl fatty acid esters obtained either from animal fat or used frying oil. Moreover, to improve the overall oxidation stability and cold flow properties of camelina derived fatty acid methyl esters, additives such as the antioxidant ionol (500 ppm) and effective depressants like Wintron XC-30 and Infineum R-442 (optimal dosages of 1500 ppm and 1200 ppm, respectively) can be added.^88^

Zhao *et al.* (2015), studied the production of camelina derived biofuel through the catalytic cracking process. They optimized the operation conditions (liquid hourly space velocity, temperature, and oil extraction press frequency) in a fixed bed tubular reactor containing a Zn/ZSM-5 catalyst and evaluated the impacts on the yield and quality of biofuel. It was observed that the oil extraction press frequency parameter significantly affected hydrocarbon biofuel production, while the liquid space velocity was the least important factor. A rection temperature of 550 °C, an oil extraction press frequency of 15 Hz, and a liquid hourly space velocity of 1.0 per hour were found to be the optimum parameters for upgrading camelina oil.^76^

Xu *et al.* (2019), also reported *ex situ* catalytic cracking of camelina oil at 450 °C in a fixed bed reactor loaded with a nickel MCM-41 catalyst. The results of this study showed that use of a Ni loaded MCM-41 catalyst improved the selectivity, yield and liquified product's chemical composition without showing any influence on the crystalline structure of MCM-41. This nickel loaded MCM-41 catalyst was found to facilitate the alkylation, isomerization, cyclization, deoxygenation, aromatization, and cracking reactions.^98^ Catalytic cracking of camelina oil for biofuel production in the presence of a non-catalyst or a ZSM-5 catalyst supplemented with various concentrations of zinc in a fixed bed reactor was investigated by Zhao *et al.* (2015). The study revealed that a ZSM-5 catalyst containing 20% zinc (wt/wt) provided the best quality and highest yield of hydrocarbon biofuel. Moreover, hydrocarbon biofuel produced by the ZSM-5 catalytic cracking displayed improved physico-chemical properties as compared to that produced by the non-catalytic cracking of camelina oil. This study also indicated that addition of Zn to ZSM-5 can possibly facilitate reactions like dehydrogenation and decarboxylation.^99^

Bastante *et al.* (2015), reported the production of biodiesel from camelina oil *via* transesterification using ultrasound as an alternative energy source and optimization of the reaction parameters. Fatty acid methyl ester content was found to meet the EN 14103 standards, and no significant difference was observed between the yields obtained by sonicated and non-sonicated mediated transesterification. However, it was determined that the ultrasound assisted transesterification required less energy and catalyst, shorter reaction times and lower reaction temperatures.^100^ Another study reported by Yang *et al.* (2016), studied the optimization of biofuel production by transesterification of unrefined camelina oil, and compared it with biodiesel yields from refined or unrefined canola oil. Temperature, catalyst, reaction time and molar ratio of methanol to oil were evaluated using a face-centered, central composite design. The highest yields of biodiesel obtained under optimum conditions were 95.6%, 95.2%, and 97.7% for unrefined camelina oil, unrefined canola oil, and refined canola oil, respectively.^101^

Boichenko *et al.* (2020), also reported the ethanolic transesterification of camelina oil using an alkaline catalyst and commercial fuel ethanol with high hygroscopicity. It was found that the presence of moisture up to 1% in the alcohol resulted in a reduction of esters yield from 71% to 58% and that the final reaction products became more contaminated with waste reaction byproducts. However, a higher ester content (92–93.5%) in the final reaction products of transesterified camelina oil was obtained. The physico-chemical properties of these camelina oil ethyl esters were reported to be more similar to those typically found in jet fuel and so could potentially serve as an alternative renewable fuel for aviation.^102^

Sun *et al.* (2015), reported the production of camelina oil derived biodiesel using supercritical alcohol mixtures to improve quality and yield. A mixture of different alcohols (*e.g.*, ethanol : 1-butanol and methanol : 1-butanol) and a range of molar ratios were studied to determine their impact on free fatty acid alkyl ester yield, the physical properties of the biodiesel and the impact of specific temperatures, pressures, and reaction times on the composition of their blend mixtures. It was concluded from this study that a molar ratio for ethanol : 1-butanol in the range of 0.5–0.7 generated the maximum yield of 85.60% of biodiesel having the lowest pour point of −14 °C, while a methanol : 1-butanol mixture with a molar ratio of 0.5–0.9 produced a maximum yield of 86.14% with a pour point of −12 °C.^103^

**Fig. 4:**
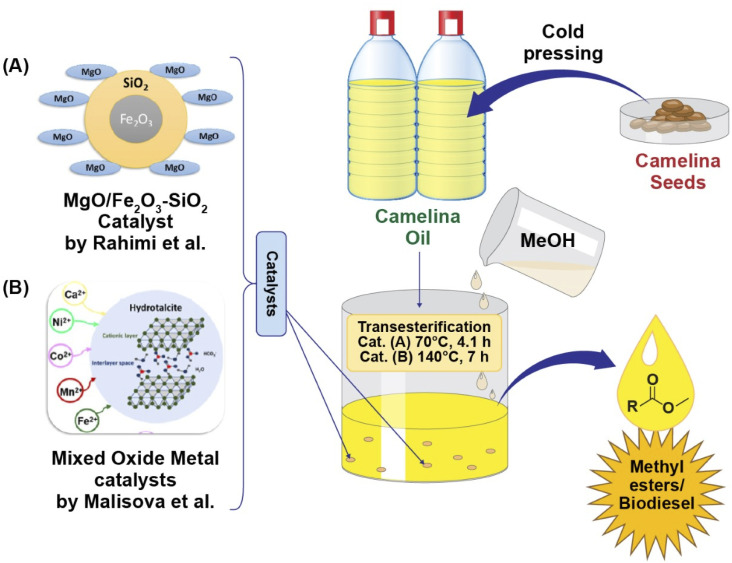
Graphical illustration of *Camelina sativa* oil methanolic transesterification catalysed by novel heterogenous catalysts (A) MgO/Fe_2_O_3_–SiO_2_ obtained from Mg (NO_3_)_2_ and Fe_2_O_3_–SiO_2_*via* precipitation method, providing fatty acid methyl esters yield of up to 99% as reported by Rahimi *et al.* 2021,^105^ and (B) mixed oxide catalyst containing different divalent cations prepared by coprecipitation of divalent cations (Mn^2+^, Co^2+^, Ca^2+^, Fe^2+^, Ni^2+^) salt solution, and solution of NaHCO_3_ and NaOH giving maximum 90.1% yield of fatty acid methyl esters as investigated by Malisova *et al.*, 2020.^96^

Malisova and coworkers (2020)^104^ performed methanolic transesterification of camelina oil catalyzed by novel mixed oxides which were developed from Mg/Al hydrotalcites added with divalent metals (Ni, Co, Ca, Mn, and Fe). A noticeable relationship between both cationic type and catalytic activity was observed during the transesterification reaction. Two of the five mixed oxide catalysts (containing Ni or Fe) provided higher catalytic activity, with a methyl ester content greater than 96.5 wt% obtained at a temperature of 140 °C after seven hours of transesterification. These yields fulfilled the minimum amount needed to meet the European standard (EN 14214). Furthermore, this study also revealed a large reduction in the amount of leached metal, particularly the Ni-mixed oxide, making it more suitable candidate for inclusion into the structure of the metal oxide/hydrotalcite. Hence, transesterification of camelina oil in the presence of a heterogenous catalyst containing a suitable combination of cations can provide a high content of methyl esters as illustrated in Fig. 4.

Biodiesel production form camelina oil using a MgO/Fe_2_O_3_–SiO_2_ core–shell magnetic nanocatalyst as reported by Rahimi *et al.* (2021)^105^ is shown in Fig. 4. The catalyst was prepared by calcination at 650 °C for 2.29 h. A fatty acid methyl ester yield of up to 99% was obtained using suitable conditions (70 °C, 4.1 h) which were optimized using a central composite design based on the response surface methodology. The developed catalyst could be reused up to 4 cycles. The results revealed that the property values of biodiesel produced form camelina oil are within the specified limits of standards, thus suggesting the ability of camelina oil as a raw material for biofuel.

Recently, Rokni *et al.* (2022) reported on the use of microwave assisted transesterification of camelina oil to produce biodiesel and optimization of the microwave reaction parameters to produce higher yields of fatty acid methyl esters. Catalyst loading, reaction time and the molar ratio of methanol to oil were assessed for their impact on yield using an RSM-BBD design. The highest biodiesel yield of 95.31% (desirable 95%) was attained under optimal conditions (molar ratio of 6.91, reaction time 5.85 min, and catalyst loading of 1.26%). Moreover, the physicochemical properties of camelina oil derived biodiesel met the standard biodiesel requirements of EN 14214, and ASTM D6751.^82^

### Biopolymers and bio-composites preparations

Development of polymeric materials and composites from an epoxidized camelina oil intermediate has been explored for various industrial applications. Balanuca *et al.* (2014), synthesized methacrylated camelina oil by reacting methacrylic acid with the oxirane rings of epoxidized camelina oil (Fig. 3), which was further copolymerized with ethylene glycol derivatives and reinforced with different polymerizable nanostructured POSS (polyhedral oligomeric silsesquioxane) molecules. The developed hybrid materials were investigated for use as a biomaterial for various biological applications.^106^

Balanuca and his coworkers (2015) explored camelina oil after its epoxidation for polymeric materials or networks and composites preparations. In one of their reports, they did partial ring opening of the epoxy ring with methacrylic acid, followed by conjugation of unreacted epoxy groups with different molecular weight hydrophilic polyethylene glycol units to develop polymerizable monomers (Fig. 3). The prepared monomers were polymerized using visible or UV radiation to produce novel polymeric networks with enhanced thermal stability.^107^ In another report, these authors manufactured interpenetrating polymer networks after methacrylation of epoxidized camelina oil. For this purpose, a blend of various ratios of methacrylated camelina oil, polyethyleneglycol dimethacrylate and epoxy resin (diglycidylether of bisphenol A) were polymerized simultaneously. The outcome of this study suggested a synergistic effect between methyacrylated camelina oil and epoxy monomers that improved the properties of the resulting polymeric materials, thus making them a great alternative to existing materials for specific end-use applications.^108^

Camelina oil derived hybrid nanocomposites as potential substitutes for non-renewable fossil oil derived conventional resins were also investigated. For this purpose, curing of epoxidized camelina oil was performed using different epoxy ring containing POSS molecules. The incorporation of POSS molecules with eight epoxy rings provided superior properties giving no aggregates and a single-phase morphology. These hybrid nanocomposites had improved thermal and mechanical properties which was attributed to reinforcement of the POSS molecules with the triglycerides of camelina oil.^109^

Kasetaite and his coworkers (2014) synthesized camelina oil derived biophosphonate crosslinked polymers and compared their thermal, mechanical, biodegradation, hydrolysis, swelling and bio-resistance properties to linseed oil derived polymers. The results revealed that the properties of camelina oil derived biophosphonate crosslinked polymers were comparable to those polymers derived from linseed oil, thus making them a promising starting material for the development of polymers.^110^

### Coatings and adhesives

Due to higher content of unsaturated fatty acids, epoxidized camelina oil has high potential to be utilized for industrial applications in the area of coatings, adhesives, lubricants, and resins. Epoxidized triglycerides from camelina have been functionalized in various ways to develop useful monomers and/or polymer precursors (see Fig. 3). Kim *et al.* (2015) reported on the epoxidation of camelina oil with formic acid and hydrogen peroxide while optimizing reaction time, temperature and catalyst ratio and concluded its potential in the field of coatings, pressure sensitive adhesives and resins.^54^ Derivatization of camelina oil into dihydroxy fatty acids *via* epoxidation is also reported in the literature as potential polymer precursors for the synthesis of bio-lubricant base stock.^111^ Moreover, ring opening of fully epoxidized camelina oil by reacting with a range of different alcohols to develop oleochemicals with potential application as bio-lubricant base stocks has also been investigated.^112^ Derivatization of camelina oil by partial epoxidation using *in situ* performic acid followed by hydroxylation with an alcohol in the presence of acid catalyst is carried out to replace castor oil polyols.^113^ Chemical functionalization of fully epoxidized camelina oil into partially acrylated triglycerides followed by their hydroxylation to develop dihydroxyl acrylated camelina oil, an acrylic polyol with one acrylate functional group, has also been shown to produce polymerizable materials.^114^

Kim *et al.* (2015), investigated the use of processed dihydroxylated camelina oil for adhesive applications. They found that formulation of dihyroxylated epoxidized camelina oil with epoxidized soybean oil by UV polymerization resulted in an oil with higher peel adhesion properties.^54^ Another research group also reported the synthesis of pressure sensitive adhesive coatings from camelina oil after its epoxidation. The epoxidized camelina oil was partially acrylated and then hydroxylation was carried out to obtain an acrylic polyol. The acrylic polyol derived from camelina oil was copolymerized with 2-ethylhexyl acrylate using ultraviolet radiation to produce viscoelastic polymers. The newly developed polyols demonstrated promising adhesive properties that could lead to replacements of similar petrochemical derived compounds.^114^

Li and co-workers (2018) reported on the synthesis of fully acrylated camelina oil *via* epoxidation and investigated its potential application in the manufacture of wood coatings and UV (ultraviolet) curable clear films. They reported high mechanical strength, glass transition temperature, gloss value, cross-linking density, and coating performance for fully polymerized acrylated epoxidized camelina oil as compared to polymerized epoxidized camelina oil. They also that, when compared to with soybean oil derivatives, camelina oil derivates exhibited enhanced potential for coating applications due to high monomer functionality. Hence, chemical modification of camelina oil into epoxidized and acrylated camelina oil derivatives adds value to the base oil feedstock making it a promising substitute for petroleum derived counterparts used for the development of UV curable coatings.^115^

Kalita *et al.* (2018), developed a range of camelina oil based poly(vinyl ether)s by varying the composition of the plant oil and the molecular weight of its components and compared these to similar molecules obtained from linseed oil and soybean oil. The developed poly(vinyl ether)s were crosslinked under suitable conditions through an autoxidation process to produce surface coatings on both steel substrates and as free standing films. Decreases in viscosity and increases in tensile strength, glass transition temperature, and Young's modulus for crosslinked networks occurred as the unsaturation level of the plant oil increased. Overall, the results from this study illustrated that manipulation of polymer molecular weight and parent plant oil selection can help to tailor the viscosity and other properties of crosslinked films developed from these plant oil based poly(vinyl ether)s, while enhancing their suitability for applications in surface coatings.^116^

Another research group prepared and studied novel, flexible bio-based acrylate coatings from cardanol modified fatty acids from camelina oil. For this purpose, in addition to the cardanol modified acrylated fatty acids of camelina oil, acrylates of camelina, cardanol and soybean were also synthesized individually followed by their epoxidation and acrylation. The developed acrylates were polymerized under UV radiation to produce bio-based acrylate coatings. It was concluded that coatings developed from the cardanol modified fatty acids of camelina oil demonstrated a higher glass transition temperature, tensile strength, maximum degradation temperature, hardness, and resistance to solvent when compared to the coatings from epoxidized acrylated soybean oil. This suggests these new types of acrylates may be promising alternatives to soybean oil derived epoxidized acrylates.^117^

Omonov and coworkers (2017) explored the potential of camelina oil derived polyols to replace those derived from castor oil for polyurethan applications because of castor oil's limited production and instabilities in supply and price. For this purpose, controlled or partial epoxidation of camelina oil followed by its hydroxylation with an alcohol was carried out. Polyols synthesized from either camelina, or castor oil were treated with diphenylmethane diisocyanate to obtain polyurethanes. The authors reported highly improved thermomechanical properties for cured polyurethanes from camelina oil derived polyols compared to those made from castor oil, thus demonstrating that camelina oil derived polyols were excellent substitutes for castor oil polyols in the production of polyurethane.^113^

Bustamante and his research group (2019), synthesized polyhydroxyalkanoates (PHAs) as renewable and biodegradable polyesters from camelina oil using *Pseudomonas* Sp. Medium chain length PHAs have shown promising properties as adhesive and elastic types of polymers. Various strains of *Pseudomonas* Sp. were shown to utilize camelina oil extremely well and produced high yields of medium chain length PHAs without any pretreatment of the raw oil.^118^

Development of bio-based hydroxy fatty acids by chemical modification of epoxidized camelina oil was reported by Sharma *et al.* (2019). Camelina oil was fractionated to remove the saturated fatty acids. The remaining oil had an enhanced iodine value (160 g_I_2__/100 g) due to the rich content of unsaturated fatty acids. These isolated unsaturated fatty acids were epoxidized and hydroxylated by acid catalyzed ring opening of the epoxides and were found to serve as a great renewable substitute for synthetic lubricant base stocks.^111^ In addition to this, Sharma and coworkers (2021), in their recent report again investigated the potential of camelina oil as a bio-lubricant base stock. Ecofriendly oleochemicals (a range of different alkoxy fatty acids) were prepared from camelina oil by conjugating presynthesized epoxidized camelina oil with various alcohols such as *n*-butanol, 2-propanol, 2-ethylexhanol, and isoamyl alcohol and their physico-chemical properties were evaluated. The authors observed interesting properties for the camelina derived alkoxy fatty acids which suggested their great potential for low temperature applications as bio-lubricant base stocks and good alternatives for synthetic and mineral oil derived lubricants.^112^

In a recent report by Mauro and coworkers (2021),^119^ they reported on the synthesis of reprocessable resins derived from epoxidized camelina oil when using antagonistic structures, either aromatic or aliphatic amines or acids, as hardeners. They explored two different combinations: the first was a combination of aliphatic/aliphatic carboxylic acids (3,3′-dithiodipropionic acid, and 2,2′-dithiodibenzoic acid), while the second consisted of only aromatic structures with acid/amine functionality (4-aminophenyl disulfide, and 2,2′-dithiodibenzoic acid). It was observed that thermosets prepared through dual crosslinking required decreased temperatures for curing and reprocessing when compared to ones obtained with individual crosslinking only. The results from this study showed the potential of camelina oil derived thermosets to produce materials with strong solvent resistance and recyclability.

### Alkyd resins

Alkyd resins are oil-based polyesters, prepared by reacting polyols, dicarboxylic acids or their anhydrides with fatty acids (Fig. 3), that have found applications in paints, varnishes, castings, and coatings *etc.* Commercially developed alkyd resins for the paint industry can be utilized in gypsum plasters, cement, metals, wood, or wood-based materials. Combining linseed or soybean oil with synthetic precursors such as phthalic anhydride or pentaerythriol was reported to produce alkyd resins. However, these synthetic raw materials are not environmentally friendly due to their toxicity^101,102^ and there is high demand to use alternatives such as non-food fatty acids (vegetable oils), as a raw material to produce alkyd resins. In this respect, camelina oil represents an excellent alternative raw material for synthesis of these materials.

Hanna *et al.* (2016), reported on one pot synthesis of alkyd resin derived from the reaction of renewable camelina oil with polyglycerol, where alcoholysis of oligomerized glycerol with camelina oil was performed. The alkyd resins were synthesized by polycondensation of anhydrides of maleic acid and phthalic acid with the alcoholysis products derived from camelina oil using temperatures of 230–250 °C. The developed alkyd resin displayed drying times and flexibility quite similar to those materials prepared or derived from pentaerythritol-camelina oil.^120^ In addition to this, Hanna and coworkers (2016) also reported on the development of alkyd resins using camelina oil as a raw material and glycerol as a polyol and their comparison with linseed oil derived materials. To prepare these alkyd resins, alcoholysis of vegetable oil with glycerol was carried out, and then esterification of selected anhydrides (maleic anhydride and phthalic anhydride) with the intermediate products from the first step was performed to produce monoesters of dicarboxylic acids. These were then subjected to polycondensation to obtain the desired end products. The results revealed that the camelina oil derived alkyd resins had comparable properties to resins derived from the more expensive linseed oil-based materials.^121^

### Agrochemicals

Agrochemicals are comprised of chemical substances and are utilized to protect and enhance the growth and fertility of crops. Various synthetic agrochemicals are being used in modern agriculture for enhancing production of crops while fulfilling the growing global demand for food.^122^ However, the historic, unsystematic use of these synthetic agrochemicals caused numerous issues like pest resistance, negative impacts on consumer's health, and contamination of air, water, and soil. To overcome these issues, development of plant derived pesticides, which are less hazardous to the environment and humans, could be a potential green alternative to synthetic agrochemicals as they are biodegradable, cheap, environmentally friendly, and can have new modes of action.^123^ Plant derived agrochemicals are utilized in various ways, for instance as powder from crude plant materials, or as pure plant materials or extracts formulated into suspensions or solutions. Numerous classes of natural compounds such as fatty acids, aldehydes, glycolipids, alcohols, aromatic phenols, terpenoids, ketones, alkaloids, flavonoids, naphthoquinones, limonoids, polyol esters, saccharides, and saponins are already reported in the literature as having or contributing to, pesticidal activity.^124–127^ Among plants specifically, camelina can also be implemented as a potent insecticide or pesticide for the growth of field crops. Hu *et al.* (2011), reported the addition of camelina meal at 1–5% (w/w) to soil to suppress the germination and hyphal growth of the fungi *Phymatotrichopsis omnivore*.^128^

Pernak and coworkers (2018) developed camelina oil derived bio-ionic liquids and investigated their use for agricultural applications. Bio-ionic liquids were prepared by reacting camelina oil with quaternary ammonium cations having different structures such as: choline, benzalkonium, di(hydrogenated tallow)dimethylammonium, tetramethylammonium, oleylmethylbis(2-hydroxyethyl)ammonium, didecyldimethylammonium or tetradecyltrimethylammonium to investigate their effect on biological properties. These bio-ionic liquids demonstrated notable differences in their activity when tested as an antifeedant treatment against storage pests such as *Trogoderma granarium* Ev., *Tribolium confusum* Duv., and *Sitophilus granarius* (L.). The prepared bio-ionic liquids also displayed high activity when tested as an adjuvant for a group of sulfonylurea herbicides. The outcome of this study emphasized that development of third generation bio-ionic liquids from cheap and renewable resources like camelina oil, has great potential to be implemented for the manufacture of novel agrochemicals.^129^

### Cosmetics

In addition to various other industrial applications, the role of vegetable oils in the cosmetic industry is also very important and they are used on a large scale because of their distinct, natural properties. They typically have little to no toxicity and are compatible with human physiology with reduced allergenicity. Moreover, they exhibit potential for use in skin protection and reactivation. Camelina oil contains bioactive compounds with antioxidant properties and is currently used in the manufacture of different cosmetics such as creams, bar soaps, balms, and lotions. Although, the current use of camelina oil is low in the cosmetic industry, it is a cheap feedstock and so has great potential to replace synthetic and natural antioxidants in cosmetic formulations.^130–133^

Natalita *et al.* (2015), investigated the antioxidant activity of different vegetable oils like camelina, safflower, hemp, rose hip, flax, and thistle for their application in cosmetics and therapeutics. High antioxidant activity was observed for camelina oil, but for cosmetic formulations hemp oil also displayed promising results.^130^

## Life cycle analysis (LCA) of *camelina sativa*

Life cycle analysis (LCA) is a process to assess the environmental impacts of a product during its life cycle starting from raw material to final disposal or recycling. A LCA of any product involves various stages for its environmental impact assessment starting from its production, extraction, processing, manufacturing, transportation, utilization, and disposal or recycling.^134^ Considering the LCA of camelina oil, only a few studies have been reported and those mainly focused on emissions of greenhouse gases (GHG) and or carbon footprint while evaluating several different parameters. Li *et al.* (2014)^81^ studied the environmental impact of camelina oil derived hydro-processed renewable jet fuel and biodiesel with regard to global warming potential, energy resource consumption, ecosystem, human health and quality. Li *et al.* (2014)^81^ also evaluated its life cycle analysis based on agricultural production in the region of Canadian Prairies, oil extraction and fuel conversion. The study found GHG emissions from 1 MJ (megajoule) of camelina derived hydro-processed renewable jet fuel varied between 3.06–31.01 kg CO_2_ per MJ equivalent and for camelina biodiesel between 7.61–24.72 g CO_2_ equivalent. However, consumption of non-renewable energy for camelina hydro-processed renewable jet fuel and biodiesel was found to be 0.13 to 0.52 MJ/MJ and 0.40–0.67 MJ/MJ, respectively. The outcome of this study signified the importance of camelina oil as a potential environmentally attractive feedstock for fuels accounting for lower GHG emissions in comparison to other fuels derived from oil seed and petroleum resources. Shonnard *et al.* (2010)^135^ investigated the GHG emissions and energy demand needed to produce green biodiesel and HRJ derived from camelina oil. This LCA considered camelina cultivation requirements, oil recovery on a commercial scale and the process of refining the crude oil. The study found GHG emissions of 18.0 and 22.4 g CO_2_ equiv. per MJ for green diesel and HRJ respectively which represents a reduction of up to 80% GHG compared to the petroleum counterparts.

Krohn and co-worker (2012)^136^ conducted a life cycle analysis of camelina biodiesel using the spread sheet model KABAM (Krohn's Alternative Biodiesel Analysis Model). They found camelina to be an environmentally viable option which generated lower GHG and reduced fossil fuel use of up to 60% when compared to petroleum derived biodiesel. Moreover, by avoiding the land use change emissions, camelina biodiesel has shown an even greater reduction in GHG emissions when compared to traditional canola and soybean derived biodiesel.

## Current challenges and future perspectives

**Fig. 5:**
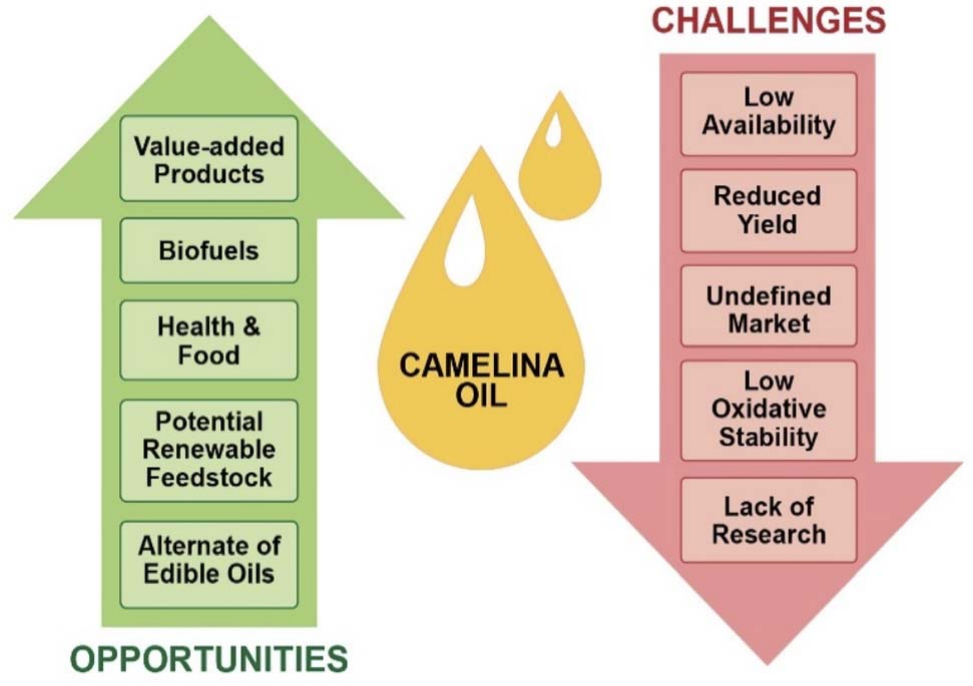
A graphical illustration calling attention to the dearth of research, poor yield, higher oxidation, and an unclear market of camelina oil as a current challenge and highlighting opportunities of camelina oil as an alternative potential feedstock of edible oils for its valorization into value added products, biofuel, health, and food.

To reduce dependency on natural resources and encourage sustainable production of materials from renewable resources, the bioeconomy concept is rapidly growing in many countries. Although the use of oilseed crops has gained huge recognition, there is considerable resistance to using edible food oils for these purposes. As discussed in this review, *Camelina sativa* has emerged as a promising nonfood oil seed crop with great characteristics over other traditional oilseed crops. However, it still has some challenges that hinder its acceptability as a global industrial oilseed crop. Major challenges include reduced yield, poorly defined markets, low profitability, low oxidative stability, and lack of knowledge and awareness amongst farmers. Moreover, its availability and continuous supply in purified form are other restraints for its application in the advancement of biofuels, fine chemicals, and value-added products. An illustration of the major challenges and possible opportunities is given in Fig. 5.

Limited research has been conducted on camelina production which still needs to be fully optimized. Improved cultivation practices, development of high yielding varieties with better seed quality and improved pest resistance may help camelina develop as a promising renewable feedstock for the biofuel, food and value-added products markets. Although more attention has been given to exploit camelina oil for biofuel production, there is a need to increase the efficiency of its processing in a cost-competitive and sustainable manner by adhering to green chemistry principals (*e.g.*, avoiding the use of toxic chemicals and minimizing processing energy demands). Furthermore, few studies have been performed on camelina oil to explore its potential in packaging, composites, polymeric materials, cosmetics, pharmaceuticals, nutraceuticals, and functional food applications. However, the composition of camelina oil, which is rich in unsaturated fatty acids, makes it an excellent feedstock for chemical functionalization to develop renewable building blocks for various industrial applications (Table 3).

**Table 3:** Challenges for camelina oil valorization

**Cultivation practices**
The current high demand for camelina oil for biofuel preparations and other industrial uses requires increased production of the camelina crop. For this purpose, improvement in cultivation practices and development of high yielding varieties is essential, which will enhance its competitiveness compared to other common oil seed crops
**High polyunsaturated contents**
The highly unsaturated fatty acid content particularly α-linolenic acid, of camelina oil makes it more susceptible to oxidation, which limits its application in biofuel production and food, and other industrial uses. The physio-chemical properties of biodiesel are strongly influenced by the chain length and degree of unsaturated fatty acid methyl esters in the initial feedstock
The presence of higher levels of α-linolenic acid in biodiesel make it incompatible with EN 14214 specifications and adversely affected the biodiesel properties such as oxidation stability, distillation temperature, the iodine value, the cetane number and the linolenic acid methyl ester content.^85^ These drawbacks can be overcome by reducing the molecular weight and unsaturated fatty acid content of biodiesel feedstock
**New valorization techniques**
In addition, emphasis on designing energy efficiency, implementing greener processes using safer solvents, preference of catalytic reagents over stoichiometric ones, and cost-effective ways should be considered to produce biofuel, organic chemicals, and value-added products

Through additional research, camelina oil has the potential not only to replace edible food oils, but also as a renewable alternative feedstock in the advancement of numerous scientific and engineering applications. For example, development of unexplored monomers from this renewable feedstock can offer numerous value-added products, while increasing its potential for commercialization and contributing to the benefit of farmers, companies, and economy.

## Conclusion

Camelina is a promising non-food, energy crop with low fertility input requirements and numerous benefits. Camelina seed is rich in oil and protein. The oil contains a high proportion of highly polyunsaturated fatty acids (particularly ω-3 fatty acid) and is also a valuable source of natural antioxidants like flavonoids, tocopherols, phenolic acids, and flavonoids. These unique characteristics make camelina a potential renewable feedstock for industrial scale production of commercial biofuel, value-added products, and potentially as an edible food ingredient. Among the multiple end-use applications of camelina oil, the key focus has been on the production of biofuel that has a lower carbon footprint compared to minerals and other plant oil derived fuels. However, more innovative research is needed to improve its performance characteristics. Through further research, unexplored monomers from camelina fatty acid triglycerides can offer potential bio-derived functionalities as building blocks for the development of sustainable polymers. In addition to the agricultural industry, extensive research efforts are required to utilized camelina oil as a promising feedstock in the production of bio-based products such as films, adhesives, coatings, plastics, composites, lubricants, cosmetics, packaging, and polymeric materials. Research progress in these sectors will increase the market for camelina oil beyond biofuels, accelerate its economic viability and lead it on the road to wider acceptance as a commercial oil seed crop.

## Author contributions

Project conceptualization, methodology, administration, funding acquisition and supervision, A. K. M. and M. M.; methodology, investigation, data analysis, writing—original draft preparation, M. A.; writing—review and editing, A. K. M., M. M., R. V. A., R. R., J. T., H. K., M. A. All authors contributed to the discussion, review and approval of the manuscript for publication.

## Conflicts of interest

The authors declare that they have no known conflict of interest that could have appeared to influence the work reported in this paper.

## Supplementary Material
